# Life history strategies complement niche partitioning to support the coexistence of closely related *Gilliamella* species in the bee gut

**DOI:** 10.1093/ismejo/wraf016

**Published:** 2025-02-02

**Authors:** Chengfeng Yang, Benfeng Han, Junbo Tang, Jiawei Hu, Lifei Qiu, Wanzhi Cai, Xin Zhou, Xue Zhang

**Affiliations:** Department of Entomology, College of Plant Protection, China Agricultural University, 100193 Beijing, China; Sanya Institute of China Agricultural University, 572024 Hainan, China; Department of Entomology, College of Plant Protection, China Agricultural University, 100193 Beijing, China; Department of Entomology, College of Plant Protection, China Agricultural University, 100193 Beijing, China; Department of Entomology, College of Plant Protection, China Agricultural University, 100193 Beijing, China; Department of Entomology, College of Plant Protection, China Agricultural University, 100193 Beijing, China; Department of Entomology, College of Plant Protection, China Agricultural University, 100193 Beijing, China; Department of Entomology, College of Plant Protection, China Agricultural University, 100193 Beijing, China; Sanya Institute of China Agricultural University, 572024 Hainan, China; Department of Entomology, College of Plant Protection, China Agricultural University, 100193 Beijing, China

**Keywords:** honeybee, species coexistence, interspecific competition, *r/k* selection, gut environmental dynamic

## Abstract

The maintenance of bacterial diversity at both species and strain levels is crucial for the sustainability of honey bee gut microbiota and host health. Periodic or random fluctuation in diet typically alters the metabolic niches available to gut microbes, thereby continuously reshaping bacterial diversity and interspecific interactions. It remains unclear how closely related bacteria adapt to these fluctuations and maintain coexistence within the bee gut. Here, we demonstrate that the five predominant *Gilliamella* species associated with *Apis cerana*, a widely distributed Asiatic honey bee, have diverged in carbohydrate metabolism to adapt to distinct nutrient niches driven by dietary fluctuation. Specifically, the glycan-specialists gain improved growth on a pollen-rich diet, but are overall inferior in competition to non-glycan-specialist on either a simple sugar or sugar-pollen diet, when co-inoculated in the bee host and transmitted across generations. Strikingly, despite of their disadvantage in a high-sugar condition, the glycan-specialists are found prevalent in natural *A. cerana* guts. We further reveal that these bacteria have adopted a life history strategy characterized by high biomass yield on a low-concentration sugar diet, allowing them to thrive under poor nutritional conditions, such as when the bee hosts undergo periodical starvation. Transcriptome analyses indicate that the divergence in life history strategies is attributed to gene expression programming rather than genetic variation. This study highlights the importance of integrative metabolic strategies in carbohydrate utilization, which facilitate the coexistence of closely related *Gilliamella* species in a changing bee gut environment.

## Introduction

Honey bees host a distinctive and specialized gut microbiome comprised of only 5–9 bacterial genera [1]. However, extensive taxonomic diversity has evolved at both species and strain levels and remained persistent within the guts of individual bees [2–5], contributing to the functional variety of the gut microbiome [5–8]. The loss of bacterial diversity is normally associated with elevated pathogen susceptibility and bee mortality in hives, as well as multiple adverse effects on honey bee metabolism, development, and the capacity in degradation of toxic substances [7–15]. Therefore, the maintenance of bacterial diversity is crucial for sustainable gut function and ecology, which are essential for bee health [16–18].

Closely related species generally metabolize the same carbohydrates [19, 20] and are, therefore, unlikely to coexist in high densities in the same gut due to competitive exclusion [21–24]. This conflicts with the notion that closely related bacterial species frequently occur in the same gut of bees [2–4], a pattern resembling that of humans [25, 26]. Niche partitioning theory has been widely applied to explain the coexistence of closely related bacteria in the gut microbiome [21, 27, 28]. For instance, niche separation can be accomplished in multiple ways [21, 23, 29], such as variations in metabolic capacity [21, 22, 30], and divergence in resource preference [29, 31–33]. However, most existing studies have focused on the mechanisms of bacterial coexistence under constant or defined diet conditions [30, 34, 35]. Such scenarios have deviated from the natural situation where animal diet constantly changes in type and abundance, which may directly affect bacterial interactions and coexistence outcomes.

Honey bees, as important pollinators, are specialized in a carbon-rich diet containing pollen and nectar, which plays a pivotal role in shaping both bacterial interactions and the community structure of the bee gut microbiome [36]. Contrary to the common perception that the diet of honey bees is simple and consistent, the overall quantity and relative proportion of pollen and nectar constantly change according to the honey bee’s age, caste, and external environment (e.g. seasonal turnover) [37, 38]. For example, nurse bees maintain a pollen-rich diet for proteins and lipids, echoing their social role in nutrient provision to the queen and larvae, whereas foragers are biased toward a nectar-rich diet to meet the quick and extensive energy requirement during foraging [39, 40]. Correspondingly, compositional variance has been identified in the gut microbiome between forager and nurse honey bees at both species and strain levels [41–44], and during winter and summer seasonal turnovers [45]. However, it remains unknow how closely related bacterial species have managed to coexist and maintained the diversity in the changing gut environment.

Despite hosting an overall similar gut microbiome as observed in the western honey bee (*Apis mellifera*), *Apis cerana* maintains host-specific gut bacteria at both species and strain levels [46]. Among the core gut bacterial genera, *Gilliamella* is specialized in pectic glycan degradation and saccharide fermentation [47], and has diverged into five predominant species in the eastern honey bee *A. cerana*, a dominant endemic Asiatic species and a key pollinator for local floras. Here, based on 61 *Gilliamella* isolates covering all species associated with *A. cerana,* by integrating evidence from multi-omics, growth dynamics, *in vitro* competition and transgenerational *in vivo* passaging, we explored the mechanisms underpinning the coexistence of closely related *Gilliamella* species in the dynamic bee gut environment. We reveal that the five *Gilliamella* species have diverged in glycan metabolism and balanced trade-offs between growth rate and yield in utilizing common sugar substrates. By employing these varied strategies, in particular, by balancing trade-offs between growth rate and growth yield, different *Gilliamella* species are capable of adapting to distinct dietary conditions, which constantly fluctuate in the bee gut environment. Our findings pave the way for understanding mechanisms underlying the maintenance of bacterial diversity in broad gut microbiome systems.

## Methods and materials

### Genome and phylogenomic analyses

A total of 200 *Gilliamella* genomes were downloaded from the NCBI genome database (Dataset S1). The full-length coding sequences were predicted using prodigal [48] and annotated using Prokka (version 1.13) [49]. The 316 single-copy core genes (shared by ≥99% strains) were identified using Roary (−blastp 75, version 3.13.0) [50] and aligned with MAFFT (version v7.467) [51]. The concatenated genes were used to construct a maximum-likelihood phylogeny using RAxML (version 8.2.12; option “-m GTRGAT”) [52], which was visualized using iTOL (version 6.8.1) [53]. Pairwise genome-wide average nucleotide identity (ANI) values were calculated using pyani (version 0.2.10; option “-m ANIb”) [54]. The pairwise gene content similarity was calculated using Jaccard coefficient in R (version 4.2.3: https://www.R-project.org/) [55] based on the pangenome-based matrix produced by Roary [50].

### Metagenomic analysis

A total of 201 gut metagenomes generated from *Apis cerana* worker bees from China (BioProject PRJNA705951) and Japan (PRJNA598094) were download from NCBI. Raw sequence reads were filtered using fastp (version 0.13.1) [56] and FastQC (v0.11.9; https://www.bioinformatics.babraham.ac.uk/projects/fastqc/). The reads were mapped to the *A. cerana* genome (GCF_001442555.1_ACSNU-2.0) using BWA aln (version 0.7.17, −n 1) [57] to identify host sequences, whereas mapped reads were filtered out by samtools (version 1.9) [58] and Picard (version 2.25.5; https://broadinstitute.github.io/picard/). Relative abundances of *Gilliamella* species were determined using the script “run_midas.py species” in MIDAS [59] with default parameters.

### Reconstruction of the carbohydrate metabolic pathways

Gene clusters (CGCs) containing carbohydrate active enzymes (CAZymes) were annotated using dbCAN (v2.0.11) [60]. Genes involved in simple sugar utilization were predicted using hmmscan implemented in HMMER (version 3) and Hidden Markov Models constructed using genes retrieved from MetaCyc [61]. Protein sequences were analyzed using kofam_scan v1.3.0 (https://github.com/takaram/kofam_scan), the command line version of KEGG [62]. The carbohydrate metabolism pathways were constructed through manual curation (see Supplementary Materials and Methods).

### *In vitro* cultivation of *Gilliamella* strains

Each of the type strains that represent the five *Gilliamella* species (GA1_B2776, GA2_B2889, GA3_B3801, GA4_B3172, and GA5_B3788) was individually struck on a heart infusion agar (HIA, Thermo Scientific) plate supplied with 5% sheep blood (Solarbio, Beijing, China). The plates were incubated at 35°C and 5% CO_2_. After 48 hours, the cells were scraped from the plates and washed with 1× phosphate-buffered saline (PBS) for three times before resuspension in PBS to a final optical density of 1 at 600 nm (OD_600_). The bacterial solution was diluted to 1/100 using fresh carbohydrate-free HIA (cfHIA) medium (Hopebio, Qingdao, China) supplemented with a defined carbon source at 10 mM, equivalent to the glucose concentration in standard HIA medium used for honeybee gut bacterial cultures. The cells were cultivated at 35°C and 5% CO_2_ in a volume of 200 μl in a 96-well plate (Corning, NY, USA). The cell density was measured using a SpectraMax i3x multi-function reader (Molecular Devices, CA, USA) at OD_600_. All growth assays were conducted in technical triplicates and repeated with three independent trials. The nonparametric smoothing spline in the R package of “growthrates” (version 0.8.4, https://CRAN.R-project.org/package=growthrates) [63] was used to determine the maximum growth rate μ (μmax).

For the *in vitro* competition experiments, the fluorescence strains and the GA1_B2776 CGC2 mutant were constructed as previously described [64] (see Supplementary Materials and Methods, and Table S2). The fluorescence intensity was measured using a SpectraMax i3x multi-function reader to quantify the bacterial growth. The level of interaction between GA1_B2776 and GA5_B3788 was calculated based on the difference in area under growth curves (AUC) between mono- and co-culture according to a previously published method [65]. The interaction strength was calculated by the function of log10 (AUC [co-culture]/AUC [mono-culture]). A Student’s t-test was used to calculate the significance of the interaction.

### Bacterial inoculation of microbiome-depleted bees

Late-stage pupae were removed manually from capped brood cells (obtained from a healthy *A. cerana* colony at an apiary in Changping, Beijing) and placed in sterile plastic boxes. The pupae were incubated for 2 days at 35°C and 50% humidity until eclosion. The newly emerged microbiome-depleted bees were transferred into plastic cup cages (20–25 bees per cup). Bacterial cells were suspended in sucrose syrup (50%, w/v) to a cell density of 10^7^ per ml and then used to feed the microbiome-depleted bees on Day 1 (1 ml inoculum solution per cup). The bees were then reared using defined diets at 35°C and 50% humidity. For each treatment, three cup cages of bees were set up as biological replicates. At least three individuals were taken from each cup replicate and used in subsequent analyses, bringing the total bee numbers to 9–30 bees for each condition. DNA was extracted from the whole gut using a CTAB-based method as previously described [1]. The relative abundance of each strain included in co-colonization was quantified using amplicon sequencing of the *frr* gene following our previous study [66]. The total bacterial number was determined by the qPCR method (Taq Pro Universal SYBR qPCR Master, Vazyme Biotech, Nanjing, China) and primers specifically targeting the 16S rRNA gene of the *Gilliamella* associated with *A. cerana* [4] (Table S1). The absolute abundance of each *Gilliamella* species (GA1–5) was then obtained by multiplying the relative abundance of each species by the total number of *Gilliamella* cells detected by qPCR according to previously published methods [67, 68].

For the gut microbiome passage experiment, three guts from bees under the same treatment were pooled and homogenized in 25% glycerol and stored at −80°C. The gut homogenate was spun down (3500 × *g* for 5 min) to remove glycerol, resuspended in 1 ml of sucrose syrup, and then fed to newly emerged microbiome-depleted bees. The inoculated bees were reared for 5 days before bacterial quantification. The abundances of GA1_B2776 and GA5_B3788 in co-colonization were determined using qPCR with primers targeting strain-specific single-copy genes (Table S1). The process was repeated for each passage until the 3^rd^ generation of microbiome-depleted bees, when the GA1_B2776 strain could no longer be detected by qPCR in some of the tested bees.

### Quantifying *Gilliamella* abundances in natural honey bee guts

Forty-eight adult worker bees were collected in one apiary in Qinghai (Dataset S3). Bees were chilled at 4°C for 10 min. The entire guts were dissected from the abdomen using sterile forceps and stored at −80°C. The relative abundances of each *Gilliamella* species in the gut were quantified using amplicon sequencing of the *frr* gene [66].

### RNA extraction, sequencing and transcriptomic analyses

Three independent mono-cultures of GA1_B2776 and GA5_B3788 were collected at the exponential growth phase. The total RNA was extracted using the Bacterial RNA Kit (Omega, USA) following the manufacturer’s instruction. RNA was purified using probes to remove ribosomal RNA. Fragmentation was carried out using divalent cations under elevated temperature in a fragmentation buffer. The strand-specific libraries were prepared as described previously [69] and sequenced using the Hiseq (PE150) platform (Illumina, CA, USA) at Novogene Bioinformatics Technology Co., Ltd (Beijing, China).

Raw sequence reads were filtered using Trimmomatic (v.0.39) [70], and aligned to reference genomes (genomes of GA1_B2776 and GA5_B3788, Dataset S1) using bowtie 2 (v.2.3.4.1; −mismatch 2) [71]. Reads were assigned to genes using featureCounts (v2.0.3) from the SubRead suite [72], and then the FPKM (Fragments Per Kilobase per Million) of each gene was calculated to estimate the gene expression level. Differentially expressed genes were analyzed using the R package “DESeq2” (v.1.20.0) [73]. The resulting *P* values were adjusted using the Benjamini and Hochberg’s approach for controlling the false discovery rate. Genes with an adjusted *P* < 0.05 and |log2[Fold Change]| ≥ |1| were considered as differentially expressed (DEGs, Dataset S4). KEGG functional enrichment analyses for the DEGs were performed using the package “clusterProfiler” (v.3.18.1) [74], with a *P* value adjustment cut-off of 0.05 as the false discovery rate.

### Statistical analyses

All statistical analyses were performed using R (version 4.2.3). The pairwise comparison was analyzed using the Mann–Whitney-Wilcoxon test and Student’s t-test, with *P* < .05 considered as being significant.

## Results

### Genome discrete *Gilliamella* species are typically co-present in the bee gut

**Figure 1:**
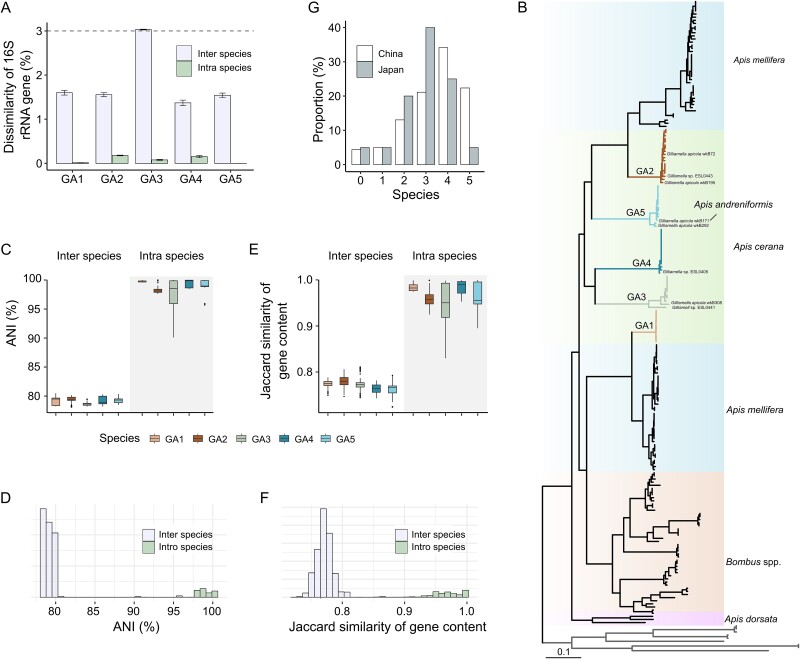
**The *Gilliamella* strains associated with *Apis cerana* diverge into five species**. (**A**) the dissimilarity of 16S rRNA gene sequence of inter and intro species of GA1–5 (*Gilliamella* species associated with *A. cerana*). (**B**) whole-genome phylogenetic tree of *Gilliamella* strains derived from *Apis dorsata*, *Bombus* spp., *A. mellifera,* and *A. cerana*. Nine genomes of the family Orbaceae were selected as outgroups (see methods). (**C**, **D**) box plot (C) and histogram (D) show the pairwise average nucleotide identity (ANI) of inter and intro *Gilliamella* species of GA1–5. (**E**, **F**) box plot (E) and histogram (F) depicting the Jaccard similarity coefficient based on pairwise gene content of inter and intro *Gilliamella* species of GA1–5. (**G**) percentage of *A. cerana* individuals across China and Japan harboring multiple *Gilliamella* species.

To investigate the species diversity of *Gilliamella* associated with the Asian honey bee *A. cerana*, sixty-one *Gilliamella* strains were isolated from 22 bees covering seven geographic populations across China (see Dataset S1). These isolates were sequence-discrete for the 16S rRNA gene, but all shared a > 97% identity (Fig. 1A). The genome-wide phylogeny placed them into five distinct clades (GA1–5) (Fig. 1B) [4]. Inter-clade isolates shared low genome-wide pairwise average nucleotide identity (ANI, ~80%) and gene content similarity (Jaccard similarity, 70–80%) (Fig. 1C-F). In contrast, intra-clade genomes showed reduced divergences: > 99% 16S rRNA gene similarity, up to 5% differences across the orthologous genomic region, and up to 10% variations in gene contents (Fig. 1C-F). Based on the broad consensus ANI divergence value applied in bacterial species delineation (> 5%–6%) [2, 75] and the phylogenetic relationships, these five *Gilliamella* clades can be classified as distinct species, and hereafter denoted as GA1 to GA5 in the present study.

Based on publicly available metagenomic data, we examined the distribution of the 5 *Gilliamella* species among *A. cerana* bee individuals across China (n = 161) and Japan (n = 40). Results showed that these *Gilliamella* species coexist persistently in the gut of *A. cerana*, with varied species numbers and combinations found in different geographic populations of the host (Fig. 1G and Fig. S1).

### *Gilliamella* species are mostly consistent in simple sugar utilization but diverged in glycan metabolism

**Figure 2:**
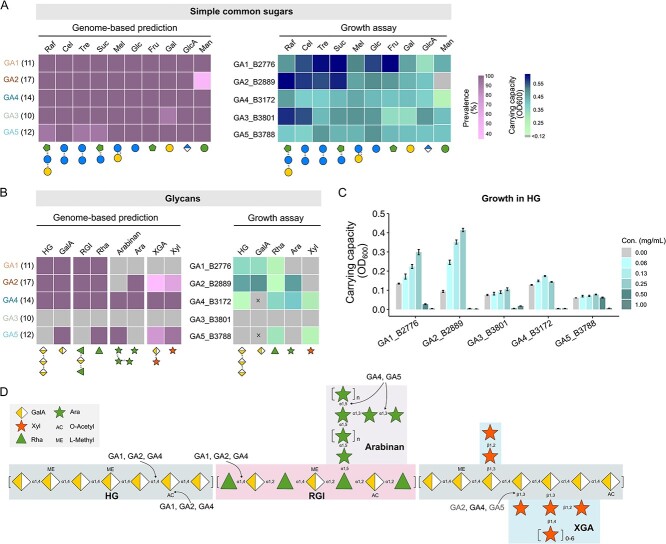
***Gilliamella* species are highly similar in simple sugar metabolism but diverged in glycan utilization.** (**A**, **B**) the occurrence of common sugar (A) and glycan (B) metabolism genes among strains of each *Gilliamella* species (GA1–5) (left panel), and the carrying capacity of the type strains of each *Gilliamella* species cultured in a carbohydrate-free heart infusion agar (cfHIA) medium containing 10 mM of the defined sugar as carbon source (right panel). The bacterial biomass is measured by optical density at 600 nm (OD_600_) and averaged from three biological replicates. Grey boxes indicate the absence of genes and OD_600_ < 0.12. Check Dataset S2 for complete carbohydrate utilization gene profiles. Raf, raffinose; Cel, cellobiose; Tre, trehalose; Suc, sucrose; Mel, melibiose; Glc, D-glucose; Fru, D-fructose; gal, D-galactose; GlcA, D-glucuronic acid; man, D-mannose; HG, homogalacturonan; GalA, D-galacturonic acid; RGI, rhamnogalacturonan I; Rha, L-rhamnose; Ara, D-arabinose; XGA, xylogalacturonan; Xyl, D-xylose. (**C**) the carrying capacity of the representative *Gilliamella* strains cultured in cfHIA medium with a gradient concentration of HG. (**D**) the representative constituents of pectic polysaccharides that are potentially accessible to *Gilliamella*.

Carbohydrate metabolism represents a critical factor in determining the structure of bacterial community [30, 76–79]. Therefore, these *Gilliamella* genomes were compared for the repertoire of genes encoding for CAZymes and sugar utilization. Genomic analyses indicated that the five *Gilliamella* species shared the ability to utilize a majority of diet-derived simple sugars, including raffinose (Raf), cellobiose (Cel), trehalose (Tre), sucrose (Suc), melibiose (Mel), D-glucose (Glc), D-fructose (Fru), D-galactose (Gal), D-glucuronic acid (GlcA) and D-mannose (Man), all of which are present in nectar or as building blocks of glycans in pollen (Fig. 2A and Dataset S2). These sugars are referred to as common sugars hereafter. The genomic prediction was further validated by *in vitro* cultivation using representative strains of each *Gilliamella* species (GA1_B2776, GA2_B2889, GA3_B3801, GA4_B3172 and GA5_B3788) on the carbohydrate-free HIA medium (cfHIA) supplied with a defined common sugar (Fig. 2A and Fig. S2).

CAZyme analyses indicated that *Gilliamella* is specialized in the degradation and utilization of certain pectin-derived glycans (Fig. 2B). However, not all *Gilliamella* species were capable of pectic glycan degradation (Fig. 2B and Dataset S2), echoing the pattern found in *Apis mellifera* [7]. Homogalacturonan (HG) and rhamnogalacturonan-I (RGI) represent two primary polysaccharides constituting the pectic backbone (Fig. 2D) [80]. Genes relevant to HG and RGI utilization (organized as CAZyme gene clusters, CGCs, Fig. S3A, B) were enriched in species GA1, GA2, and GA4 (Fig. 2B and Dataset S2). RGI may contain arabinan side chains, and the HG backbone linked with D-xylose side chains becomes xylogalacturonan (XGA) (Fig. 2D) [80]. Species GA4 was also characterized by genes relevant to Arabinan and XGA utilization, including CAZymes of families GH43_10 (α-L-arabinanase) and GH43_11 (β-xylosidase) (Dataset S2). HG is predominantly found in pectin (up to 65%) [81]. Thus, using it as the representative glycan, we further tested the carrying capacity of the three *Gilliamella* strains growing in HG at gradient concentrations. Results showed that GA1_B2776 and GA2_B2889 displayed substantial growth on both HG and GalA, in a concentration-dependent manner (Fig. 2C and Fig. S3C). However, GA4_B3172 displayed limited growth on HG and did not grow on GalA even under high substrate concentrations (Fig. 2C and Fig. S3C). These results suggested that GA1 and GA2 were superior in HG utilization.

Growth assays were carried out for each strain using the cfHIA medium supplied with the building block saccharides of these glycans, including galacturonic acid (GalA), rhamnose (Rha), arabinose (Ara), and xylose (Xyl). The results were mostly consistent with genomic prediction, albeit with a few exceptions such as GA4_B3172 and GA5_B3788 with GalA (Fig. 2B, depicted in cross marks).

### Glycan degrading *Gilliamella* gain growth benefits under a pollen diet

We investigated whether *Gilliamella* strains depended on glycan to grow *in vivo*. When each of the five representative strains was mono-inoculated into newly emerged microbiota-depleted honey bees, all bacteria successfully colonized the gut and reached a high abundance with or without pollen supplement (Fig. S4). Thus, glycans were not required for the successful colonization of glycan-degrading *Gilliamella* when each of them was inoculated alone.

**Figure 3:**
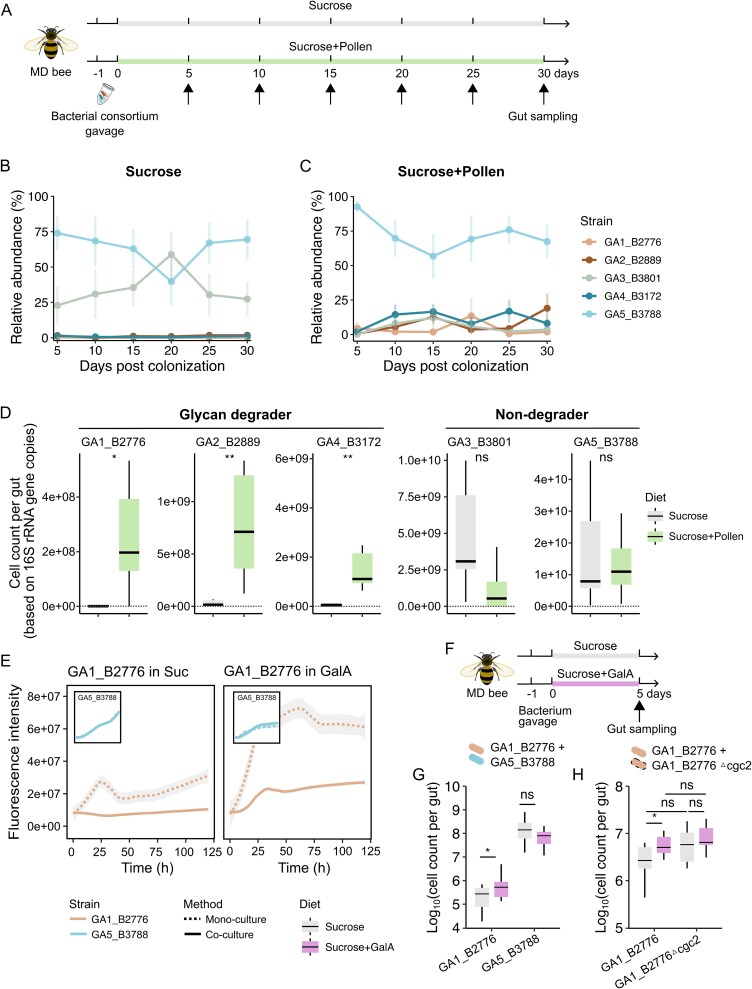
**Pollen-derived carbon sources promote the growth of glycan degrader strains.** (**A**) schematic illustration of the colonization experiment. Three cup cage replicates were set up for each diet condition. MD, microbiota-depleted. (**B**, **C**) the population dynamics of each *Gilliamella* strain under either the sucrose only (B) or sucrose and pollen (C) diet. Mean values ± SEM (vertical shading) are indicated. n = 5 ~ 6 individual bees from three cup cages for each measurement. (**D**) the absolute abundance of each *Gilliamella* strain between two different dietary regimes on Day 10. n = 5 ~ 6 individual bees from three cup cages for each measurement. ^*^P < .05, ^*^^*^P < .01; ns, not significant (Mann–Whitney-Wilcoxon test). (**E**) cell density measured by fluorescence intensity of GA1_B2776*::gfp* and GA5_B3788*::rfp* under a sucrose medium with (right panel) or without (left panel) GalA. Dash line, mono-culture; solid line, co-culture. (**F**) experimental schematic of the co-colonization of two strains in microbiota-depleted bees. (**G**) the absolute abundances of strain GA1_B2776 and GA5_B3788 under either dietary conditions. n = 14 individual bees from three cup cages per measurement. ^*^P < .05; ns, not significant (Mann–Whitney-Wilcoxon test). (**H**) the absolute abundances of the wild-type and GalA-mutant strain of GA1_B2776 on Day 5. n = 9 individual bees from three cup cages for each measurement. GA1_B2776*△cgc2*, with GalA utilization loci spanning the *exuT* and *uxaCBA* genes in CGC2 knocked out. ^*^P < .05; ns, not significant (Mann–Whitney-Wilcoxon test).

To test whether glycan degraders could benefit from additional glycan sources, we examined their changes in abundance within an artificially mixed bacterial community, which was inoculated in newly emerged microbiome-depleted bees. We mixed the five representative *Gilliamella* strains at an equal abundance and inoculated them to newly emerged microbiome-depleted bees, which were reared under either a sucrose-only diet or that supplemented with pollen (Fig. 3A). The results showed that a significant increase in both relative and absolute abundances was observed in glycan degraders upon pollen supplementation (strains of GA1, GA2, and GA4, Mann–Whitney-Wilcoxon test, *P* < .05 or *P* < .01; Fig. 3B-D), confirming that they did benefit from pollen.

To validate that the specialized glycan utilization capacity has caused the increased growth of GA1, 2, and 4 under a pollen diet, one glycan-degrader (GA1_B2776) and one non-degrader (GA5_B3788) were selected as representative strains for competitive experiments. The choice of GA5_B3788 was made based on the results from the co-colonization experiments using five *Gilliamella* strains, where it showed the highest abundance (Fig. 3B and C). An equal number of each bacterium were cultured on the cfHIA medium supplied with sucrose or galacturonate acid (GalA, the HG digestion product). Whereas GA1_B2776 alone was able to grow well in either sucrose or GalA medium, its growth was suppressed by the presence of GA5_B3788 in co-culture in sucrose, which was in turn removed in GalA supplied medium (Fig. 3E). The promotion in growth of GA1_B2776 by GalA was also confirmed *in vivo* in co-colonization with GA5_B3788 (Mann–Whitney-Wilcoxon tests, *P* < .05, Fig. 3F and G). Furthermore, when a GalA utilization mutant GA1_B2776 (the CGC2 knockout strain, Fig. S3A) competed with the wild-type in microbiome-depleted bees under either a sucrose-only diet or that supplemented with GalA, only the wild-type strain could gain an extra growth upon GalA supplementation (Mann–Whitney-Wilcoxon tests, *P* < .05) (Fig. 3H). Thus, using the simplified two-bacteria system, we validated that the exclusive nutrients from pollen conferred a growth benefit for the glycan-degrading species.

### Glycan degraders are weak competitors for common sugars

**Figure 4:**
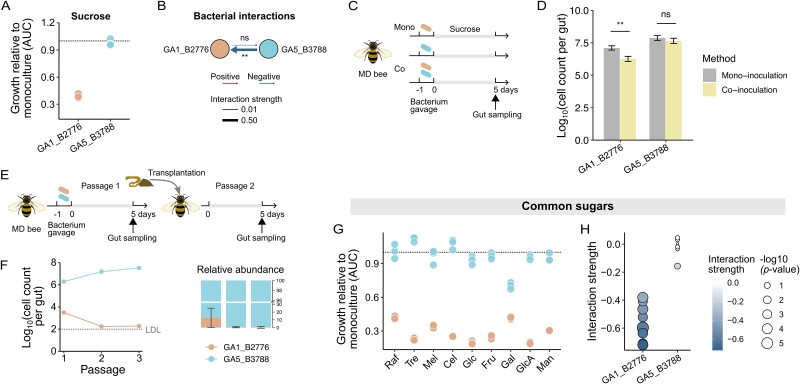
**Strain GA5_B3788 outcompetes GA1_B2776 on the common sugar substrates.** (**A**) the relative growth of GA1_B2776 and GA5_B3788 in co-culture compared to their mono-culture. Results were depicted as the area under the growth curve (AUC) in co-culture divided by the AUC in mono-culture. Dashed line represents an equal growth. (**B**) interactions between GA1_B2776 and GA5_B3788 in cfHIA medium with 10 mM sucrose. Arrow thickness represents interaction strength, which is proportional to log10 (AUC [co-culture] / AUC [mono-culture]), Student’s t-tests were used to calculate the significance. (**C**) schematic illustration of the mono-inoculation and co-inoculation of strain GA1_B2776 and GA5_B3788 in microbiota-depleted bees. (**D**) the absolute abundances of GA1_B2776 and GA5_B3788 in mono- or co-colonized bees. n = 8 ~ 10 individual bees from three cup cages for each measurement. ^*^^*^P < .01; ns, not significant (Mann–Whitney–Wilcoxon test). (**E**) schematic of the gut microbiome transplantation experiment. The homogenate of three guts was used to inoculate microbiota-depleted bees of the next generation. (**F**) the absolute and relative abundances of GA1_B2776 and GA5_B3788 over passages. Mean values ± SEM (vertical shading) are indicated. n = 16 ~ 21 individual bees from three cup cages for each measurement. LDL, limit detection line. (**G**) the relative growth of GA1_B2776 and GA5_B3788 in co-culture compared to their mono-culture in cfHIA medium supplemented with 10 mM of a defined simple sugar. Raf, raffinose; Cel, cellobiose; Mel, melibiose; Tre, trehalose; Glc, D-glucose; Fru, D-fructose; gal, D-galactose; GlcA, D-glucuronic acid; man, D-mannose. (**H**) interactions between GA1_B2776 and GA5_B3788 in cfHIA medium supplemented with 10 mM of defined simple sugar.

Despite that pollen supplement significantly improved the growth of glycan degraders (strains of GA1, 2, and 4), their relative abundances remained substantially lower than those of the non-degraders (especially GA5_B3788). This result was consistent with the observation where GA1_B2776 was inferior to GA5_B3788 in competition when co-cultured *in vitro* in the sucrose based cfHIA medium (Fig. 3E), suggesting that the glycan-degraders are weak competitors for sucrose, which is abundant in regular bee diets applied in experiments (50% sucrose syrup). Congruently, judging by the area difference under the growth curves (AUC) of the mono- and co-culture experiments, we concluded that GA5_B3788 had posed a significantly negative impact on GA1_B2776 in the sucrose medium (Fig. 4A, B). Then, an *in vivo* competition was performed and recapitulated the *in vitro* results: the colonization abundance of GA1_B2776 was significantly reduced when co-colonized with GA5_B3788 in comparison to its mono-colonization (Fig. 4D). Moreover, when the gut homogenates originated from the first-generation bees were transplanted across generations into new microbiome-depleted bees (Fig. 4E), GA1_B2776 rapidly decreased in abundance along the transmission, reaching ca. 10^3^ in passage 2 and approaching the detection limit of qPCR in passage 3 (ca. 10^2^ cells, not detectable in two of the 21 guts, Fig. 4F).

GA1_B2776 remained inferior to GA5_B3788 in competition across all common sugar substrates (Fig. 4G, H). The supernatant of the GA5_B3788 mono-culture did not inhibit the growth of GA1_B2776 (Fig. S5), suggesting that direct antagonistic interaction via toxin secretion had not likely posed a strong influence on the observed bacterial dynamic. However, we could not rule out the possible involvement of a contact-dependent antagonism between the two bacteria (e.g. mediated by the type six secretion system). A consistent competitive interaction may have contributed to the weakened colonization of GA1_B2776 when co-inoculated with GA5_B3788 under a mixed sugar condition.

### High-yield growth strategy enables glycan degraders to proliferate under low-sugar conditions

**Figure 5:**
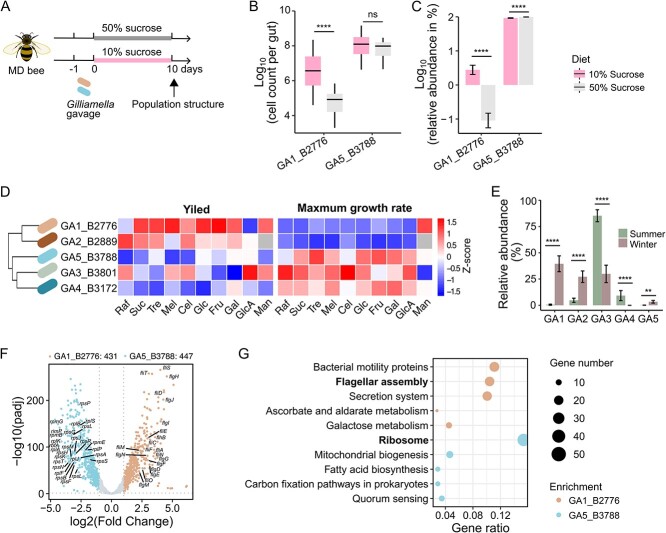
**The divergence in life history strategy allows GA1_B2776 to adapt to a low-sugar condition.** (**A**) schematic illustration of the co-colonization of strain GA1_B2776 and GA5_B3788 in microbiota-depleted bees rearing under either a 10% or a 50% (w/v) sucrose syrup. (**B**, **C**) the absolute (B) and relative (C) abundances (%) of GA1_B2776 and GA5_B3788 cultured in a sucrose solution of a high (50% w/v) or low (10% w/v) concentration measured on Day 5. The y-axis indicates the log10 value of the relative abundance of each bacterial strain under a particular sucrose condition, e.g. the value −1 represents a relative abundance of 0.1%. n = 20 ~ 30 individual bees from three cup cage replicates per treatment. ^*^^*^^*^^*^P < .0001; ns, not significant (Mann–Whitney-Wilcoxon test). (**D**) Dendrogram clustering of the *Gilliamella* type strains based on the bacterial growth traits (carrying capacity and growth rates) across all common sugar substrates. (**E**) relative abundance of *Gilliamella* species in wild bees collected in summer or winter. Bars show the mean relative abundance (%) of each strain. Error bars show ± SEM. n = 24 bees per season. ^*^^*^P < .01, ^*^^*^^*^^*^P < .0001 (Mann–Whitney-Wilcoxon test). (**F**) volcano plots showing adjusted *P* value (padj) versus gene expression fold changes between GA1_B2776 and GA5_B3788 revealed in transcriptome analysis. Genes involved in ribosome and flagellar assembly with log2(fold change) > = 2 and -log10(*padj*) > 50 are marked. (**G**) KEGG pathways significantly enriched from featured genes of GA1_B2776 and GA5_B3788. *Padj* < .05, fisher test, Benjamini-Hochberg adjustment.

The extremely weak competitivity of glycan degraders under a sugar-based diet was surprising, as it contradicted the fact that they were found prevalent in natural honey bee guts (Fig. S1). Given that the natural nutrient landscape of bees varies in both carbohydrate types and concentration (sugar content in diet ranging between 1% to 53%) [39], we thus speculated that *Gilliamella* species might have evolved to excel in varied sugar concentrations. Therefore, alternations in sugar quantity is expected to change the competitive outcomes between the two bacteria. To test this, bees co-colonized with GA1_B2776 and GA5_B3788 were subjected to a low-sugar diet (10%). The population demographics were compared to that of a regular sugar diet (50%) on Day 10 (Fig. 5A). Consistent with our speculation, the absolute abundance of GA1_B2776 significantly increased by two orders of magnitude on the low-sucrose diet, however, that of GA5_B3788 remained unchanged (Mann–Whitney-Wilcoxon test, *P* < .01; Fig. 5B). The change in cell number was also reflected in a significant elevation of relative abundance in GA1_B2776 and a corresponding reduction in the proportion of GA5_B3788 (Fig. 5C).

To further explore the mechanisms underlying the increased competitivity of GA1_B2776 under the low-sugar diet, the bacterial growth traits, including growth rate (*r*) and carrying capacity (*K*, biomass yield), were determined from the growth curves of the five *Gilliamella* strains under each of common sugars (Fig. S2). The five *Gilliamella* strains were clustered in a growth trait dendrogram (Fig. 5D). Glycan degraders (GA1_B2776 and GA2_B2889) were typically featured by a high carrying capacity, those do not degrade glycan (GA3_B3801 and GA5_B3788) and those with weak degradation capacities (GA4_B3172) had managed to maintain a relatively high growth rate under most common sugars (Fig. 5D). This echoed an *r/K* selection theory that has been implemented in broad ecosystems spanning animals, plants and microbes [82–84].

A low-quantity sugar diet can be expected in overwinter bees in temperate populations, which often undergo seasonal starvation caused by over-harvesting or climate oscillation [85]. We investigated a typical *A. cerana* population from temperate China (Guide, Qinghai Province, China) and examined changes in *Gilliamella* abundance in worker bees collected from summer and winter colonies. As expected, the relative abundance of species GA1 and GA2 (*K* strategy) significantly increased in winter bees, concomitant with the decreased abundance of the *r-*strategy species (e.g. strains of GA3 and GA4) except for GA5, whose abundance slightly increase for yet unknown reasons (Mann–Whitney–Wilcoxon test, *P* < .0001; Fig. 5E).

### Global transcriptional programming underpins the divergence in life history strategies

To gain insights into the regulatory mechanisms underlying these varied life history strategies, we compared the transcriptomes of GA1_B2776 and GA5_B3788 obtained at the exponential growth phase when mono-cultured on sucrose. The transcriptional profiles of the 1826 orthologous genes (representing 80.6% of the pangenome) differed significantly between the two bacteria (Fig. 5F, Fig. S6A). Specifically, a total of 431 and 447 genes were upregulated in GA1_B2776 and GA5_B3788, respectively (Fisher’s exact test, *Padj* < .05, Bonferroni corrected, Fig. 5F and Dataset S4). Most of the upregulated genes in GA1_B2776 were enriched in KEGG pathways associated with bacterial motility, flagellar assembly and secretion system, but the upregulated genes in GA5_B3788 were most significantly enriched in the ribosome pathway (Fig. 5G and Fig. S6A). A fast growth depends on the rate at which the cell is able to synthesize proteins, which is directly relevant to the ribosome content in the cell [86, 87]. Therefore, the upregulation of the ribosome pathway in GA5_3788 echoed its *r* strategy. In contrast, advanced mobility can improve bacterial ability to follow nutrient gradients [88, 89]. Thus, the upregulation of genes associated with motility and flagella assembly in GA1_B2776 is in line with its adoption of a *K* strategy, where an allocation of cellular resources to prioritize nutrient chemotaxis leads to a high growth yield at a cost of growth rate [90]. Consistently, using an electron microscope, we found that GA1_B2776 had a long flagella attachment on the bacterial extracellular matrix, which was absent in GA5_B3788 (Fig. S6B). The swimming assay further confirmed that GA1_B2776 was capable of moving further than GA5_B3788, despite that both bacteria carried intact flagella encoding genes (Fig. S6C). The same variation in motility was observed in between other strain pairs representing GA1 and GA5 (GA1_B2828 vs. GA5_B2824, GA1_B2840 vs. GA5_B2838, Fig. S6D). Collectively, these results reveal a divergence in regulatory strategies that allow different bacterial species to adapt distinct niches defined by carbon source quantity.

## Discussion

Elucidating mechanisms underlying the coexistence of closely related bacteria are central to research on gut microbiome diversity and function. This study aims to unravel mechanistic insight into the coexistence between closely related *Gilliamella* species under fluctuating honey bee gut environments. To address this, we isolated strains covering all five predominant *Gilliamella* species that have engaged in long-term adaptation and diversification within the gut of *Apis cerana*. We reconstructed the carbohydrate metabolic patterns of each *Gilliamella* species and conducted a thorough investigation to understand the impacts of changing diet on bacterial colonization and interaction. Our findings revealed that *Gilliamella* species have diverged in carbohydrate metabolism and become adapted to varied niches, e.g. those derived from dietary fluctuations associated with honey bee labor division or environmental factors. Whereas some *Gilliamella* species consistently show superior performance under a high-sugar diet, others have developed specialized glycan degradation capacity, which allows them to proliferate under a pollen rich diet, e.g. that of nurse bees. In general, the deviation in the life history strategy between *Gilliamella* species may allow each of them to fit a particular set of niches under drastic variations in nutrient conditions, e.g. from fully fed to starvation experienced by the bee host. As bees may undergo periodical starvation due to seasonal changes or food shortage, which could have acted as a selection force that favors *K* strategy bacteria. In particular, over winter bees tend to suffer more severe food shortage due to over-harvesting [40, 91].

From an evolutionary perspective, the concurrent metabolic profiles of the *Gilliamella* species point to an evolutionary arms race involving changes in both genetics and regulatory networks during the long-term association in the bee gut. Particularly, the glycan degraders (e.g. GA1) specialize in glycan utilization, which extends their metabolic niches on the exclusive carbon source under a pollen-supplemented diet. However, they are, in turn, less competitive in utilizing the common carbon sources that are available to all closely related bacteria, reflected in slow growth rates. In contrast, the weak glycan degraders (e.g. GA5) are superior in utilizing the common carbon sources, enabling them to quickly flourish on these substrates. Glycan degraders, to cope with exploitative competition on common carbon sources, employ a life history strategy of efficient growth that enables them to survive on a lower quantity carbon source, representing a bacterial bet-hedging during feast and famine turnover. The divergence in life history strategies is pervasive among all five closely related bacterial species, categorizing them into two clusters (GA1 and GA2 belong to the *K* type, but the other three species fall in the *r* type). Even though our study only used representative strains of GA1 and GA5 for experimental validation, similar competitive outcomes are expected for strains with similar metabolic patterns. Moreover, consistent life strategy choices for each bacterial species were observed across all common resources. We reason that due to the relatively simple dietary composition of honey bees, dietary perturbations (such as feeding cycles) might cause the abundance of different carbon sources to change in similar trends. Thus, a consistent life strategy, rather than one that is more complex and variable, may convey a cohesive selection force from multiple carbon sources on the same bacterium and facilitate metabolic niche partitioning at diet quantity gradients.

The type and frequency of the dietary disturbances can vary at time scales, ranging from hours (e.g. feeding cycle) and days (behavioral caste ontogeny) to months (seasonal transition or changing landscape). Even in the same hive, honey bees of varied labor divisions are biased in dietary types (i.e. pollen or nectar enriched), which may vary according to feeding status (dietary quantity variance), leading to altering demographic structure in bacterial community. Moreover, recent experimental and *in silico* studies have demonstrated a diversity-disturbance relationship [92], suggesting that a maximal diversity can be observed at intermediate frequencies of disturbance [93]. According to this theory, the coexistence outcomes of the closely related *Gilliamella* may have also been influenced by the frequency of dietary disturbance. The relationship between dietary disturbance frequency and strain coexistence remains untapped in this study. However, it should be mentioned that honey bees are social insects with hundreds of thousands of worker bees living in the same hive, assuring gut bacterial transmission among individuals [94, 95]. Therefore, despite that dietary disturbances that change in type and intensity may impose varied impacts on bacterial coexistence, the inter-individual bacterial transmission might have facilitated the preservation of closely related *Gilliamella* on the colony level.

As we gain in-depth knowledge on the impacts of the gut microbial community on honey bee biology, the capacity to precisely predict gut community properties becomes crucial for maintaining host health. Being able to identify how gut bacteria diverge in metabolic flexibility and how regulatory networks respond to dietary fluctuations can contribute to the understanding of how certain strains manage to coexist with their close relatives [23, 96]. Additionally, bacterial turnover at species and strain levels may change the functional traits of the honey bee gut microbiome (e.g. resistance to pathogenic microbes) and the interference with host physiology and behavior [44, 97, 98]. Here, we unravel how carbohydrate metabolism has mediated the coexistence of closely related bacteria in honey bee gut under fluctuations in nutritional landscape. The *Gilliamella* species have adjusted their carbohydrate metabolic capacities to adapt to distinct gut environments driven by dietary disturbances, promoting bacterial coexistence and maintaining taxonomic diversity, which are essential for the sustainability of gut function and host health. Our study creates a new route to predict varied bacterial responses toward nutritional changes, providing new perspectives in honey bee management.

## Supplementary Material

Supplementary_material_wraf016

Datasets_wraf016

## Data Availability

The source data generated in this study have been deposited on Dryad (https://doi.org/10.5061/dryad.cnp5hqcdv). The sequencing data for the RNA-seq and MGAS in this study have been submitted to NCBI under BioProject PRJNA1134693. The scripts for genome and metagenome analysis are available on GitHub (https://github.com/cfyang911007).
