# Supplementary material for: Life history strategies complement niche partitioning to support the coexistence of closely related Gilliamella species in the bee gut
Source: ISME J. 2025 Feb 2;19(1):wraf016. doi: 10.1093/ismejo/wraf016 (PMC11822680; doi:10.1093/ismejo/wraf016)
Supplement: Supplementary_material_wraf016 [file supplementary_material_wraf016.pdf]

Life history strategies complement niche partitioning to support the coexistence of closely related *Gilliamella* species in the bee gut

Chengfeng Yang[1,2], Benfeng Han[1], Junbo Tang[1], Jiawei Hu[1], Lifei Qiu[1], Wanzhi Cai[1],
Xin Zhou[1,2*] and Xue Zhang[1,3*]

[1]Department of Entomology, College of Plant Protection, China Agricultural University, 100193 Beijing, China

[2]Sanya Institute of China Agricultural University, 572024 Hainan, China

[3]Lead contact

*Correspondence: zhangxue05@cau.edu.cn (X. Zhang), xinzhou@cau.edu.cn (X. Zhou).

Supplementary Materials and Methods

Prediction for the carbohydrate utilization pathways

To predict the carbohydrate metabolic landscape of *Gilliamella*, we focused on three sets of genes involved in carbohydrate degradation: **a.** CAZymes and CAZyme gene clusters (CGCs). These genes were annotated using the dbCAN2 meta server using HMMER (a threshold of e-value < 1e-15, coverage > 0.35) and CGCFinder (Distance ≤ 2, signature genes = CAZyme+TC) search tool, respectively (Dataset S2) [1]. **b.** The genes involved in carbohydrate metabolism pathways, including those responsible for the metabolism of sucrose, trehalose, maltose, melibiose, lactose, L-arabinose, D-arabinose, L-fucose, D-galactose, L-galactose, L-rhamnose, D-xylose, D-mannose, D-fructose, D-glucose, L-glucose, N-Acetylneuraminate and N-Acetylmannosamine, and the glycolysis, TCA cycle, pentose phosphate pathways, were extracted from the MetaCyc database. These genes were integrated as a customized database, which contains the respective gene names, protein IDs, KO annotations, and EC assignments. Protein sequences of the genes were extracted from the NCBI or UniProt database. For each protein family, a Hidden Markov Model (HMM) was constructed using hmmbuild, and then all the HMM models were organized into an HMM database using hmmpress. *Gilliamella* genomes were searched against this HMM database using hmmscan (with the threshold of e-value < 1e-15, coverage > 0.50) implemented in HMMER (version 3) [2]. **c.** The protein sequences of the predicted CDSs were searched using KOfam against a customized HMM database of KEGG Orthologs (KOs) using KofamKOALA [3]. The KOs involved in carbohydrate metabolism (including those of glycolysis/gluconeogenesis, TCA cycle, pentose phosphate, pentose and glucuronate interconversions, fructose and mannose metabolism, galactose metabolism pathway), encoding for transporters (ABC transporters, sugar transporters of major facilitator superfamily (MFS), phosphotransferase system (PTS)) were extracted. The carbohydrate utilization pathways of *Gilliamella* were ultimately constructed based on these three gene sets. For pathways lacking particular enzyme genes, we performed a manual search based on the genome annotation to confirm the absence of the genes.

Construction of the *Gilliamella* mutant strains

Using homologous recombination as previously described [4], we created a knock-out mutant strain (GA1_B2776*Δcgc2*), by replacing the genomic regions spanning genes of

exuT, *uxaC*, *uxaB* and *uxaA* in the CGC2 (CAZyme gene cluster 2) of GA1_B2776 to a chloromycetin resistance (*cam*^{R}) gene. We also constructed three knock-in strains (GA1_B2776::*gfp*, GA5_B3788::*rfp*, expressing GFP and RFP protein, respectively), by inserting the fluorescent gene *gfp* and *rfp* into a 132-bp intergenic region between the genes *thiI* and *cpxR* in the genome of GA1_B2776 and GA5_B3788, respectively. The fluorescent gene was linked with a kanamycin resistance (*kan*^{R}) gene as selection markers. All primers and plasmids used in this study were listed in Tables S1 and S2, respectively. For mutant selection, 20 μg/mL of kanamycin (Kan), or 10 μg/mL of chloromycetin (Cam) were applied.

Knockout: Two flanking homology sequences of ~500 bp were PCR amplified from GA1_B2776 genomic DNA with primers containing the BsaI recognition site and the 4 bp overhangs [4]. The *cam*^{R} gene was amplified from pBTK301 [4]. All amplifications were carried out using the high-fidelity DNA Polymerase (2 × Phanta Max Master Mix; Vazyme, Nanjing, China) and were purified using an E.Z.N.A.® Gel Extraction Kit (Omega, GA, USA). Each PCR products (upstream homology, downstream homology and *cam*^{R} gene) was Golden Gate assembled with corrected connector parts (pYTK002 and pYTK067, pYTK004 and pYTK072, pYTK003 and pYTK068, respectively) into pYTK095 backbone containing the ColE1 origin [5] using BsaI-HF®v2 and T7 DNA Ligase (New England Biolabs, MA, USA). The three obtained plasmids were ultimately assembled into the shuttle plasmids pBTK599s in a connector-dependent order using BsmBI-v2 (NEB) and T7 DNA Ligase.

Fluorescent protein-coding gene insertion: As described above, the flanking homology sequences of ~500 bp were PCR amplified from GA1_B2776 or GA5_B3788 genomic DNA. The *gfp* and *rfp* genes, both driven by the PA3 promoter, were amplified from plasmid pBTK555 and placI_RFP (kindly provided by Barrick Lab), respectively. The *kan*^{R} gene was amplified from plasmid pBTK519. The fluorescence gene (*gfp* or *rfp*) was linked with the *kan*^{R} gene by homologous recombination using overlap extension PCR based on a 30 bp overhang. The homologies, fluorescence genes, *kan*^{R} gene, and the connector parts (pYTK002 and pYTK072) were Golden Gate assembled into the backbone vector pBTK401 (pMMB67EH *ori oirT*). The obtained plasmid was further assembled into the shuttle plasmids pBTK599s using BsmBI-v2. The obtained suicide plasmid with a R6K

origin was then used for inserting *gfp*/*rfp* into GA1_B2776 or GA5_B3788 via conjugation and recombination.

Electroporation: The assembled pBTK599s based plasmids (shuttle plasmid derivatives) were electroporated into *E. coli* MFD*pir*. In detail, MFD*pir* was grown in the LB broth with 0.3 mM DAP (meso-2,6-diaminopimelic acid; TCI Shanghai, China) at 37°C to an OD_{600} of ~0.6. Electrocompetent MFD*pir* was prepared by washing with 10% ice-cold glycerol for three times before resuspending. Each of the pBTK599s derived plasmids were electroporated individually into the competent MFD*pir* using a Bio-Rad MicroPulser Electroporator at 1.8 kV (for 1 mm cuvettes) (Bio-Rad, CA, USA). Cells were resuspended in 1 mL of LB+DAP (0.3 mM) broth and allowed to recover at 37°C for 1 h. After centrifuge at a low speed, cells were all plated on LB+DAP agar plates with respective antibiotics. Plates were incubated for 14~16 h at 37°C until the appearance of colonies.

Conjugation and recombination: The obtained strains were used as donor and incubated with *Gilliamella* GA1_B2776 or GA5_B3788 (recipient) for conjugation. MFD*pir* with specific plasmid was grown in liquid LB medium with selective antibiotics and DAP until reaching an early stationary phase. Recipient GA1_B2776 and GA5_B3788 were cultured on HIA plates for 48 h. Recipient and donor strains were washed three times with PBS, and resuspended with 100 μL of PBS. Then the cell suspensions of the recipient and donor were mixed at a 9:1 ratio of density. 100 μL of the mixture was spotted onto an HIA plate supplemented with 0.3 mM DAP prior to incubation for ~12-14 h at 35°C. The bacterial spots were scraped into a micro centrifuge tube filled with 1 mL of PBS, washed and spun down gently for two times. The conjugation mixtures were then resuspended with 1 mL of PBS. An aliquot of 100 μL of the 10-fold dilution of those mixtures were plated onto HIA plates with selective antibiotic. The plates were incubated for 2-3 days. Colonies on the plate were re-streaked on selective media. Successful construction was confirmed by amplification test for sequence spanning the target genomic region (and/or by visible fluorescence). The strains integrated with fluorescence genes were passaged on fresh HIA plates without antibiotic selection at a 48 h interval for 5 passages to test for the stability of GFP/RFP expression.

In vitro cultivation of GA1_B2776 in the spent media

The mono-culture and co-culture of GA1_B2776 and GA5_B3788 were spined down (12,000 × *g* for 10 min), and a partial of the supernatant was used to measure the pH using a standard pH meter (INESA, Shanghai, China). The remaining supernatants were sterilized using the Millex-GP filters with 0.22-μm pores (Millipore, Molsheim, France). The filtered supernatants were supplemented with a defined sterile carbon source at 10 mM to replenish the nutrients. The wild-type GA1_B2776 was inoculated into the medium supplied with the filtered culture supernatant and incubated in the 96-well plates at 35 °C and 5% CO_2. Growth was measured based on OD_{600} at a regular time-interval until the stationary growth phase. For all growth assays, three independent replicates were used, and all the experiments were repeated three times.

Motility assays and transmission electron microscopy (TEM) analysis

An aliquot (1 μL) of the bacterial solution (OD_{600} = 1) of GA1_B2776 or GA5_B3788 was inoculated onto HIA agar plates (with 0.4% or 2% w/v agar). These plates were incubated at 35 °C and 5% CO_2 for 48 h. The expansion diameter was measured with a ruler. For the transmission electron microscopy visualization, an aliquot (5 μL) of the bacterial solution was added dropwise into a copper mesh with a carbon film and dried at room temperature. The film was negatively stained for 2 min using 1% phosphotungstic acid (pH 7.4) before the remaining solution was blotted with a filter paper. Flagellar morphology was visualized using transmission electron microscopy (JEM-2000EX, JEOL, Japan).

References

1. Zhang H, Yohe T, Huang L, Entwistle S, Wu P, Yang Z, et al. dbCAN2: a meta server for automated carbohydrate-active enzyme annotation. Nucleic Acids Res. 2018;46:W95–W101.
2. Potter SC, Luciani A, Eddy SR, Park Y, Lopez R, Finn RD, et al. HMMER web server: 2018 update. Nucleic Acids Res. 2018;46:W200–W204.
3. Aramaki T, Blanc-Mathieu R, Endo H, Ohkubo K, Kanehisa M, Goto S, et al. KofamKOALA: KEGG Ortholog assignment based on profile HMM and adaptive score threshold. Bioinformatics. 2020;36:2251.

4. Leonard SP, Perutka J, Powell JE, Geng P, Richhart DD, Byrom M, et al. Genetic Engineering of bee gut microbiome bacteria with a toolkit for modular assembly of broad-host-range plasmids. ACS Synth Biol. 2018;7:1279–1290.
5. Lee ME, DeLoache WC, Cervantes B, Dueber JE. A Highly Characterized Yeast Toolkit for Modular, Multipart Assembly. ACS Synth Biol. 2015;4:975-986.

Supplementary Figures

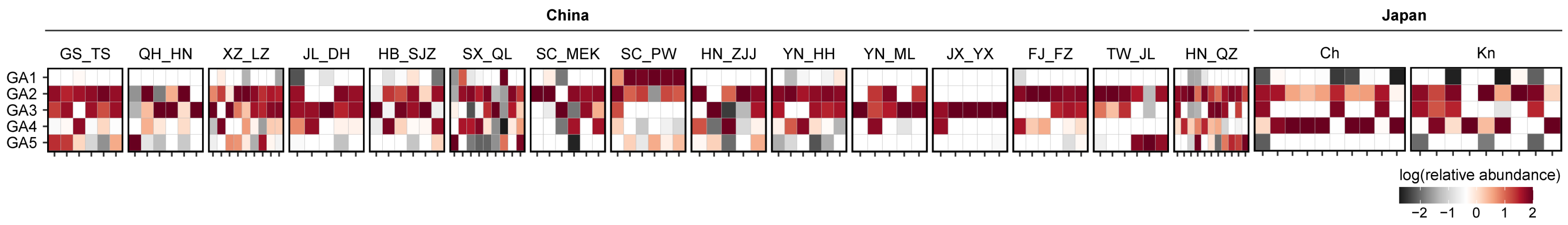

Fig. S1. Component and co-occurrence of *Gilliamella* species in *Apis cerana* gut microbiome. Heatmap depicting the relative abundance of the *Gilliamella* species in individual bees from different geographic populations in China and Japan. The raw metagenomic sequences of these samples were downloaded from BioProjects PRJNA705951 (China) and m (Japan) under the NCBI database, and in which the details of sampling sites are included.

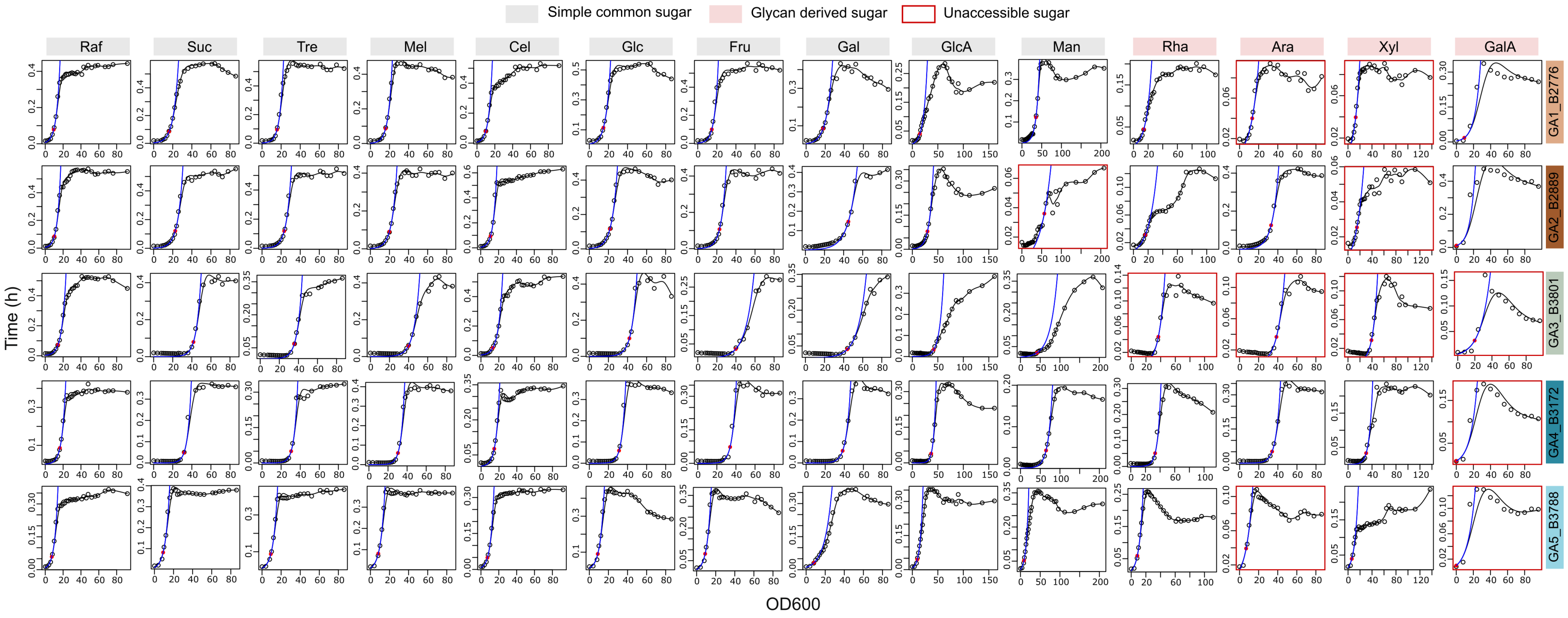

Fig. S2. Growth curves of the type strains of each *Gilliamella* species. Strains are cultured in carbohydrate-free HIA (cfHIA) medium containing 10 mM of the indicated simple sugar as carbon source. Growth is monitored by optical density at 600 nm (OD_{600}) measurements. n = 3 technical replicates per condition. Black curves show the spline fit from the R package 'growthrates', and blue curves are the exponential functions used to determine the maximum growth rate μ_{max} for each strain. Absence of abilities for strains to utilize the indicated sugars are framed in red, the growth curves in those present the minimal growth in cfHIA.

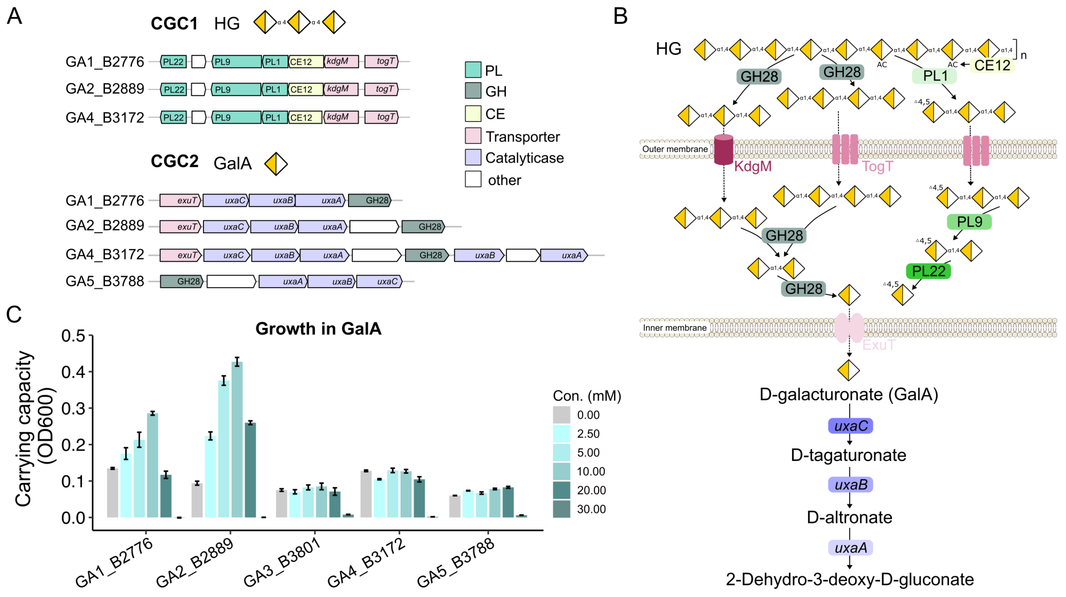

Fig. S3. *Gilliamella* species display different metabolic capability of pectic saccharides. **A** Genes involved in the digestion of homogalacturonan (HG) and its degradation products including oligo- or mono-galacturonic acids (GalA) are organized into CAZyme gene clusters (CGC1 and CGC2). CAZyme genes are categorized by functions and labeled with colors. PL, polysaccharide lyases; GH, glycoside hydrolase; CE, carbohydrate esterase. **B** A schematic picture describes a probably process of a homogalacturonan degradation and consuming by *Gilliamella.* PGA, polygalacturonic acid; AC, acetylation. **C** Carrying capacity of the type strains of each *Gilliamella* species cultured in cfHIA medium supplementing with GalA at different concentrations. Growth is monitored by OD_{600}; Mean values ± SD (vertical shading) are indicated. n = 9 biological replicates per condition.

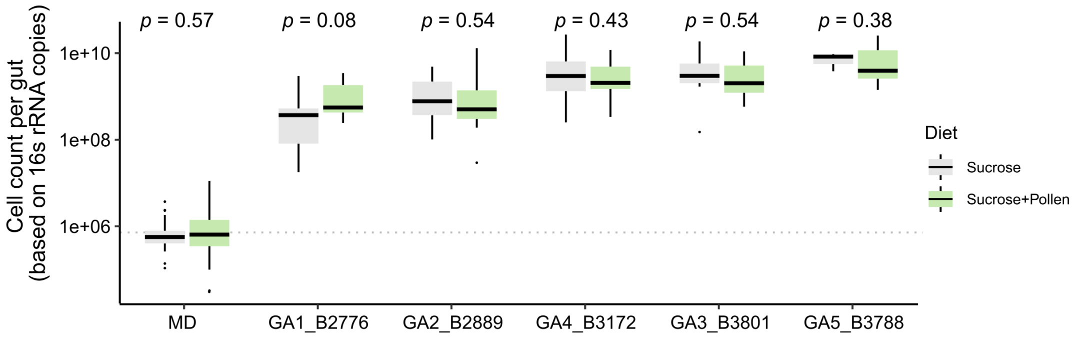

Fig. S4. All representative *Gilliamella* strains can be individually inoculated in MD bee guts, under either a sucrose-only or a sucrose and pollen diet. **MD, microbiome-depleted.** The pairwise comparison was analyzed using the Mann-Whitney-Wilcoxon test.

A

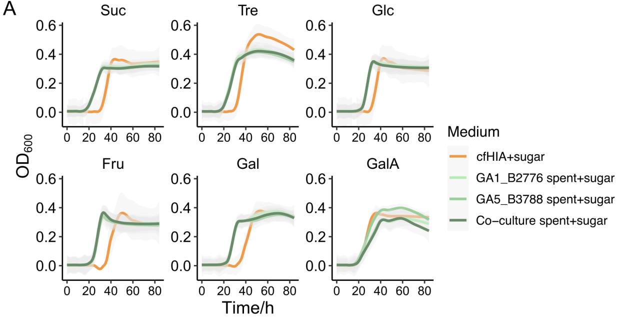

B

Sugar	Mono-culture				Co-culture	
	GA1_B2776		GA_5B3788			
	pH	△pH	pH	△pH	pH	△pH
Raf	5.87	-1.05	5.63	-1.29	5.75	-1.17
Suc	6.15	-0.86	5.96	-1.05	6.06	-0.95
Tre	6.18	-0.78	5.98	-0.98	6.05	-0.91
Mel	6.49	-0.54	6.42	-0.61	6.44	-0.59
Cel	6.45	ND	6.59	ND	6.32	ND
Glc	6.75	-0.38	6.72	-0.41	6.72	-0.41
Fru	6.66	-0.4	6.63	-0.43	6.72	-0.34
Gal	6.67	ND	6.67	ND	6.69	ND
GlcA	6.52	-0.41	6.63	-0.3	6.64	-0.29

Fig. S5 GA5_B3788 shows no obvious direct antagonistic effect to GA1_B2776. **A** Cell density of wild-type strain GA1_B2776 grown in spent media of GA1_B2776 and GA5_B3788 mono- or co-cultures. The spent media are sterilized through a 0.22-μm membrane and supplemented with a corresponding sterile carbon source at 10 mM to replenish the nutrients. Lines present logistic fit growth to time-series measurements of OD_{600} and grey shaded areas include the 95% confidence interval of three biological replicates. **B** Culture pH value changes after GA1_B2776 and GA5_B3788 growth in mono- and co-culture. Raf, raffinose; Suc, sucrose; Cel, cellobiose; Tre, trehalose; Mel, melibiose; Glc, D-glucose; Fru, D-fructose; Gal, D-galactose; GlcA, D-glucuronic acid; GalA, D-galacturonic acids.

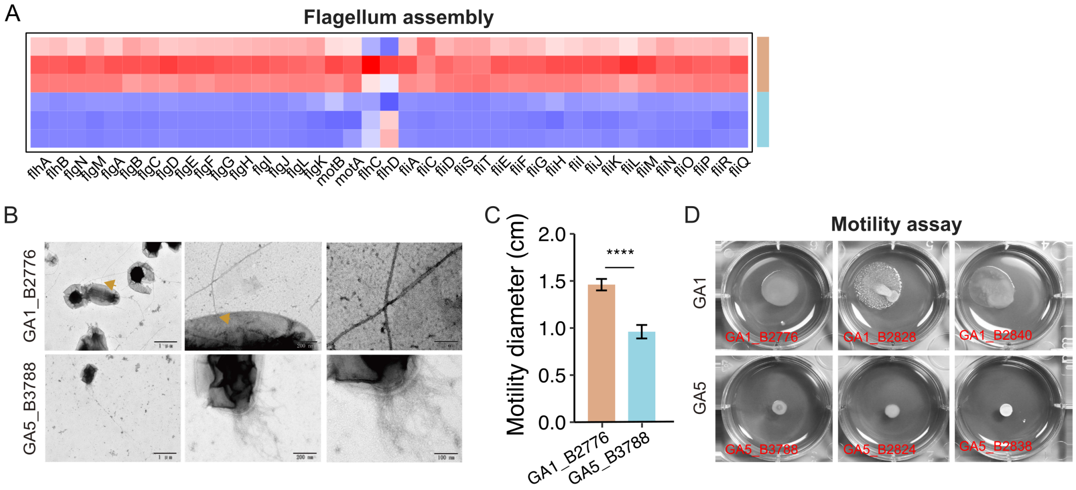

Fig. S6. The strain GA1_B2776 and GA5_B3788 show distinct transcriptional profiles and motility. **A** Heatmaps depicting the relative expression (FPKM) of genes related to flagella assembly between strain GA1_B2776 and GA5_B3788. **B** Electron micrograph of strain GA1_B2776 and GA5_B3788. Bars are 1 μm (left), 200 nm (middle) and 100 nm (right), respectively. **C** Motility diameter comparison between GA1_B2776 and GA5_B3788. ****$p < 0.0001$ (Mann-Whitney-Wilcoxon test). **D** Motility assay of strains of GA1 and GA5. Bacterium was incubated on HIA medium with 0.4 % agar and incubated for 48 h at 35°C.

Table S1. Primers used for bacterium quantification and engineering.

Name	Forward (5' to 3')	Reverse (5' to 3')	Length / bp	Role
B2776-ts	gtcGGTCTCa**aacg**gcctgagttttgtggcg	tctGGTCTCa**cata**ctgtgccgagttattatata	530	For insertion of fluorescent protein gene
B2776-tx	gtcGGTCTCa**atcc**tgtggaatcttgatgta	tctGGTCTCa**cagc**ctgactgctgttttcga	532	For insertion of fluorescent protein gene
B3788-ts	gtcGGTCTCa**aacg**gcccgaattctgtggcgtaattt	tctGGTCTCa**cata**tttgtattttcactcaatta	533	For insertion of fluorescent protein gene
B3788-tx	gtcGGTCTCa**atcc**ttttatatttaatccaa	tctGGTCTCa**cagc**aataggctgattgctgtttacg	529	For insertion of fluorescent protein gene
GFP_KanR	gtcGGTCTCa**tatg**gtgaaacaaaacggttgacaac	**tcagaattggttaattggttgtaacac**tgacaaatgctctttccctaaactcc	940	Amplifying *gfp* gene to connect with kan^R gene
mRFP1_KanR	gtcGGTCTCa**tatg**gtgaaacaaaacggttgacaac	**tcagaattggttaattggttgtaacac**cgggagagtgttcaccgacaaa	1011	Amplifying *mRFP1* gene to connect with kan^R gene
KanR_GFP	**ggagtttagggaaagagcatttgtca**gtgttacaaccaattaaccaattctga	tctGGTCTCa**ggat**ctgatccttcaactcagcaaaag	1031	Amplifying KanR gene to connect with *gfp* gene
KanR_mRFP1	**tttgtcggtgaacactctcccg**gtgttacaaccaattaaccaattctga	tctGGTCTCa**ggat**ctgatccttcaactcagcaaaag	1023	Amplifying kan^R gene to connect with *mRFP1* gene
YRFP-Kan	acctcccacaacgaagacta			Sequencing primer for verification of the correct connected *mRFP1_*kan^R
YGFP-Kan	agacacaacattgaggatggaa			Sequencing primer for verification of the correct connected *gfp_*kan^R
YR6K	ttagccatgagggtttagttcgt			Sequencing primer for verification of the correct assembly of R6K-origin-based pBTK599s suicide plasmid
YB2776	tattgctcgtcaaatcggca	gctcaacgtatctcgagatatga	3051(GFP), 1152(WT)	For verification of the correct insertion of fluorescent protein gene in GA1_B2776
YB2776US	tattgctcgtcaaatcggca	gtcgtttcatgtggtacatcgt	609	For verification of the correct insertion of fluorescent protein gene in GA1_B2776
YB2776DS	gagcaagacgtttcccgttgaa	gctcaacgtatctcgagatatga	792	For verification of the correct insertion of fluorescent protein gene in GA1_B2776
YB3788	cttagcgcggcaaattggca	accaccattctcaaggagcttaa	3091(RFP), 1119(WT)	For verification of the correct insertion of fluorescent protein gene in GA5_B3788
YB3788US	cttagcgcggcaaattggca	gtcgtttcatgtggtacatcgt	612	For verification of the correct insertion of fluorescent protein gene in GA5_B3788

Name	Forward (5' to 3')	Reverse (5' to 3')	Length / bp	Role
YB3788DS	gagcaagacgtttcccgttgaa	accaccattctcaaggagcttaa	756	For verification of the correct insertion of fluorescent protein gene in GA5_B3788
GA-ts	gtcGGTCTCa**aacg**gatggtctgttgcttatttgc	tctGGTCTCa**cagc**cattttatgctccctatac	541	For deletion of *exuT* and *uxaBCA* gene
GA-tx	gtcGGTCTCa**aacg**aaaagtggtgtaacgctataa	tctGGTCTCa**cagc**accttaggctatcacgatta	519	For deletion of *exuT* and *uxaBCA* gene
CamR	gtcGGTCTCa**aacg**accaataaaaaacgcccg	tctGGTCTCa**cagc**ttgatcgggcacgtaagag	902	Amplifying *camR* gene to assembly into plasmid pBTK095
Y095	ttacggttcctggccttttgct			Sequencing primer for verification of the correct assembly of *camR* assemblied pYTK095
Y599s	ggcatttctgtcctggctgg	ccacctatcacggaattgat	2016	Sequencing primer for verification of the correct assembly of R6K-origin-based pBTK599s suicide plasmid
YGB2776	gagttgcagcaaactcagca	cgtccaacttggagactgagt	2110(Δ*cgc2*), 6920(WT)	For verification of the correct deletion of *exuT* and *uxaBCA* gene in GA1_B2776
YUS	gagttgcagcaaactcagca	cgtaatttgatatcgagctcgctt	722	For verification of the correct deletion of *exuT* and *uxaBCA* gene in GA1_B2776
YDS	ccattgggatatatcaacggtggta	cgtccaacttggagactgagt	781	For verification of the correct deletion of *exuT* and *uxaBCA* gene in GA1_B2776
Acer_Gillia_16S	tgagtgcttgcacttgatgacg	atatgggttcatcaaatggcgca	~150	Quantifying *Giliamella* abundance
B2776spcific	ttacgtcaacaggactgcct	gacagcaagaacgatatatccgt	147	Quantifying GA1_B2776 abundance
B3788spcific	tgcgtaactgacacctaacaat	acgccacataataatcctgaagc	105	Quantifying GA5_B3788 abundance
B2776WT	caccgtcttcgaagttcataaca	gcagttccagtacggttccaaa	119	Quantifying GA1_B2776WT abundance
B2776MU	ggatgacggcaactataagaca	gtggcctaaaatatttccatcttcc	115	Quantifying GA1_B2776*Δcgc2* abundance

Note: Capitalized letters indicate enzyme recognition sequence. Letters in bold represent enzyme cutting sites.

Table S2. Plasmids used in this study.

Plasmid	Resistance	Nutrition	Golden Gate part	Role
pBTK555	SpecR	DAP		PA3, *gfp*
placI_RFP	AmpR			PA3, *mRFP1*
pBTK519	KanR	DAP		kan^R
pYTK002	CamR		Type 1	Connector
pYTK072	CamR		Type 5	Connector, cam^R
pBTK301	CamR		Type 6, 7	Linker
pBTK401	AmpR		Type 8	Entry vector for step I
pBTK599s	SpecR	DAP	Type 6, 7, 8	Entry vector for step II
pYTK067	CamR		Type 5	Connector
pYTK004	CamR		Type 1	Connector
pYTK003	CamR		Type 1	Connector
pYTK068	CamR		Type 5	Connector
pYTK095	AmpR		Type 6, 7, 8	Entry vector for step I

Note: The plasmids were kindly provided by Barrick Lab (https://barricklab.org/twiki/bin/view/Lab/WebHome).
